# Racial and Ethnic Variation in Lipoprotein (a) Levels among Asian Indian and Chinese Patients

**DOI:** 10.1155/2011/291954

**Published:** 2011-05-23

**Authors:** Dipanjan Banerjee, Eric C. Wong, Jessica Shin, Stephen P. Fortmann, Latha Palaniappan

**Affiliations:** ^1^Program on Prevention Outcomes and Practices, Stanford Prevention Research Center, Stanford University School of Medicine, Stanford, CA 94305, USA; ^2^Division of Cardiovascular Medicine, Stanford University School of Medicine, Stanford, CA 94305, USA; ^3^Palo Alto Medical Foundation Research Institute, Palo Alto, CA 94301, USA; ^4^Stanford Prevention Research Center, Stanford University School of Medicine, Stanford, CA 94305, USA

## Abstract

*Background*. Lipoprotein (a) [Lp(a)] is an independent risk factor for cardiovascular disease (CVD) in Non-Hispanic Whites (NHW). There are known racial/ethnic differences in Lp(a) levels, and the association of Lp(a) with CVD outcomes has not been examined in Asian Americans in the USA. *Objective*. We hypothesized that Lp(a) levels would differ in Asian Indians and Chinese Americans when compared to NHW and that the relationship between Lp(a) and CVD outcomes would be different in these Asian racial/ethnic subgroups when compared to NHW. *Methods*. We studied the outpatient electronic health records of 2022 NHW, 295 Asian Indians, and 151 Chinese adults age ≥18 y in Northern California in whom Lp(a) levels were assessed during routine clinical care from 2001 to 2008, excluding those who had received prescriptions for niacin (14.6%). Nonparametric methods were used to compare median Lp(a) levels. Significance was assessed at the *P* < .0001 level to account for multiple comparisons. CVD outcomes were defined as ischemic heart disease (IHD) (265 events), stroke (122), or peripheral vascular disease (PVD) (87). We used logistic regression to determine the relationship between Lp(a) and CVD outcomes. *Results*. Both Asian Indians (36 nmol/L) and NHW (29 nmol/L) had higher median Lp(a) levels than Chinese (22 nmol/L, *P* ≤ .0001 and *P* = .0032). When stratified by sex, the differences in median Lp(a) between these groups persisted in the 1761 men (AI v CH: *P* = .001, NHW v CH: *P* = .0018) but were not statistically significant in the 1130 women (AI v CH: *P* = .0402, NHW v CH: *P* = .0761). Asian Indians (OR = 2.0) and Chinese (OR = 4.8) exhibited a trend towards greater risk of IHD with high Lp(a) levels than NHW (OR = 1.4), but no relationship was statistically significant. *Conclusion*. Asian Indian and NHW men have higher Lp(a) values than Chinese men, with a trend toward, similar associations in women. High Lp(a) may be more strongly associated with IHD in Asian Indians and Chinese, although we did not have a sufficient number of outcomes to confirm this. Further studies should strive to elucidate the relationship between Lp(a) levels, CVD, and race/ethnicity among Asian subgroups in the USA.

## 1. Introduction

Lipoprotein (a) [Lp(a)] is an independent risk factor for cardiovascular disease (CVD) [1–6]. Based on its structural homology with plasminogen, Lp(a) is believed to increase CVD incidence through an increased risk of vascular thrombosis. This mechanism differs from that of other CVD-related lipids, such as cholesterol [7, 8]. Lp(a) levels are genetically determined (roughly one third of the variation in levels is explained by genetics) [9–12] and differ significantly by racial/ethnic group, for example, it is well established that African Americans have higher Lp(a) levels than Whites [13]. The distribution of Lp(a) levels in Asians in the USA is less well characterized, particularly since many studies have failed to distinguish between diverse Asian subgroups (such as Asian Indians and Chinese). With CVD rates rising in both native and immigrant Asians, characterizing the distribution of Lp(a) levels in Asian racial/ethnic subgroups and its relationship to CVD outcomes may aid efforts to reduce CVD risk in these populations [14].

The few studies that have made distinctions between racial/ethnic groups have reported higher levels of Lp(a) in Asian Indians compared with Chinese [15, 16]. However, both of these studies were performed in small volunteer samples (which may not reflect larger clinical populations) and were performed prior to standardization of Lp(a) assays, which makes their results less reliable [17–19]. Other studies have also reported higher Lp(a) levels in Asian Indians compared with Non-Hispanic Whites (NHW), but the evidence is conflicting [20–22]. Lp(a) distributions also differ by gender, [1, 4] but no studies have examined gender differences in the distribution of Lp(a) in different Asian subgroups. The association between Lp(a) levels and CVD may also be modified by race: elevated Lp(a) levels do not seem to confer the same increase in CVD risk in African Americans compared to NHW [13], and the association between Lp(a) and CVD in Chinese is equivocal [23].

Taking advantage of a large multiethnic patient cohort in a practice group that used standardized Lp(a) assays, which reduce variability in Lp(a) results, we sought to compare Lp(a) distributions in male and female Asian Indians, Chinese, and Non-Hispanic Whites. We also sought to characterize the association between Lp(a) levels and CVD in these racial/ethnic subgroups.

## 2. Methods

### 2.1. Setting

The Palo Alto Medical Foundation (PAMF) is a mixed-payer, outpatient-focused healthcare organization in the San Francisco bay area of Northern California that includes over 300 physicians serving more than 400,000 active patients with large Asian Indian and Chinese populations (both 11%).

### 2.2. Inclusion/Exclusion

We examined the electronic health records (EHR) of all adult (≥18 years) patients from January 1, 2001 to December 31, 2008. There were 2,891 NHW, Asian Indian, or Chinese patients with at least one Lp(a) result in the chart (drawn as part of routine clinical care). Approximately 15% of those with an Lp(a) were also on niacin and were excluded from the analysis since niacin lowers Lp(a) levels, leaving 2468 patients for analysis. The study received approval from the Palo Alto Medical Foundation Institutional Review Board on September 11, 2008.

### 2.3. Clinical Definitions

Low density lipoprotein cholesterol (LDL-C), high density lipoprotein cholesterol (HDL-C), total cholesterol, and triglyceride levels were obtained via the EHR and date matched to lipoprotein (a) results. Prescription for medication was used to determine medication usage as pharmacy records were not available. Cardiovascular events were defined by an ICD-9 code on a regularly maintained problem list or diagnosis from a specific patient encounter. Ischemic heart disease (IHD, ICD-9: 410.X-414.X) and cardiovascular disease (CVD, a composite of IHD, PVD, and stroke, ICD-9: 410.X-414.X, 430.X-438.X, 443.X) were defined accordingly.

## 3. Laboratory Methods

Lp(a) consists of LDL-C bound to apolipoprotein (a) via apolipoprotein B-100 in a 1 : 1 Molar ratio. Apolipoprotein (a) carries a region called the K4 type II repeat sequence, or kringle repeat, and the number of repeats of the kringle sequence is genetically determined and variable. Lp(a) levels were measured using a Quest immunoturbidmetric assay (Polymedco, USA) shown to be independent of apolipoprotein (a) size; an Lp(a) value >75 nmoL/L was considered abnormal. Other Lp(a) assays use an antibody directed at the K4 type II repeat sequence (the kringle sequence) and, thus, will tend to under- or overestimate Lp(a) concentration based on the isoform of apolipoprotein (a) used to calibrate the machine. Roughly 30% of the clinically available Lp(a) values were obtained using a Vertical Auto Profile assay (Atherotech, USA), but these results were not used due to the variability of this assay (antibody directed at kringle sequence) and the uncertainty of converting these results (mg/dL) into nmoL/L, which cannot be standardized due to heterogeneity in LDL-C particle size (apolipoprotein (a) isoform). Total cholesterol, LDL-C, HDL-C, and triglycerides were measured by spectrophotometry using a Siemens Dimension RxL analyzer (Siemens Healthcare Diagnostics, USA). HDL and triglycerides were measured via a direct method, while LDL-C was calculated using the Friedewald equation LDL-C = [TC-HDL-C-(TRIG/5)]. All laboratory measurements were carried out immediately on fresh blood samples.

## 4. Statistical Analysis

We employed a cross-sectional analysis to examine Lp(a) distributions stratified by race/ethnicity and gender. We also investigated the association between Lp(a) levels and CVD outcomes (ischemic heart disease, stroke, and peripheral vascular disease), stratified by race/ethnicity and gender.

### 4.1. Univariate

Patient characteristics were described using means, medians, and proportions. Differences in proportions were evaluated using the Chi-square test. Normality was evaluated by the quantile-quantile plots and the Shapiro-Wilk test of normality. Due to nonnormality of many of the measures, including Lp(a), nonparametric tests were used for statistical inference including Kruskal-Wallis, Wilcoxon, and Brown-Mood median tests.

### 4.2. Multivariate

We sought to determine the relationship between Lp(a) levels and CVD outcomes across race/ethnicity adjusting for known risk factors. We built logistic regression modeling CVD outcomes for each race/ethnicity. Lipoprotein (a) was divided into quintiles, and we compared the prevalence of CVD and IHD in the top quintile with the bottom four quintiles, adjusting for age, gender, and HDL and LDL levels. Total cholesterol or triglyceride levels were highly correlated with LDL and HDL levels, respectively, and thus were not included in the regression models.

### 4.3. Sensitivity

As a sensitivity analysis, we analyzed the relationship between niacin use and Lp(a) levels. We repeated the multivariate analysis on a sample including patients that were initially excluded for niacin use and included niacin usage in regression models as appropriate.

Significance was assessed at the *P* < .0001 level employing a Bonferroni correction for multiple comparisons undertaken during the analyses. All analyses were performed using SAS 9.2 (Cary, NC, USA).

## 5. Results

**Figure 1:**
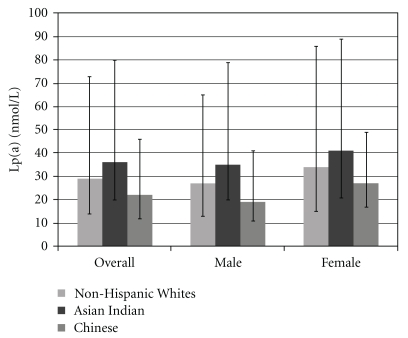
Median Lipoprotein (a) concentration by racial/ethnic group and interquartile range (25th–75th percentile).

Table 1 displays the demographic and risk factor characteristics of our cohort, stratified by gender and race/ethnicity. Approximately 1.2% of NHW, 2.0% of Asian Indians, and 0.7% of Chinese Americans had their Lp(a) determined in this clinical cohort. Most clinical parameters were similar, but Asian Indian men and women were younger and exhibited lower HDL levels than their counterparts. When comparing Lp(a) levels between NHW, Asian Indians, and Chinese (Figure 1), there was a significant overall difference in median Lp(a) levels across all groups (*P* < .0001), as well as between Asian Indians and Chinese (*P* = .0001). In contrast, there was no significant difference between NHW and Chinese (*P* = .0032) or NHW and Asian Indians (*P* = .0085).

This pattern of higher median levels of Lp(a) in Asian Indians and NHW compared to Chinese was the same for both men and women (Table 2). In women, overall differences between the groups were observed but did not achieve statistical significance at the *P* < .0001 level (Kruskal-Wallis, *P* = .1642). In men, however, the overall differences did achieve statistical significance (*P* < .0001) with Asian Indians specifically having a significantly higher median value compared to Chinese (*P* = .0001) and a higher median compared to NHW that was not quite statistically significant (*P* = .0086). Chinese had lower median values compared to NHW, but again the difference was not quite statistically significant (*P* = .0018). 

There was no significant univariate association between Lp(a) levels and CVD events (381) in any racial/ethnic group. Our multivariate analysis revealed that in the overall cohort, there was no relationship between elevated Lp(a) (5th quintile 89 nmoL/L and above) and IHD or CVD (composite outcome) after adjusting for age, gender, race/ethnicity, BMI, LDL, and HDL (data not shown). For each racial/ethnic group, having a Lp(a) value in the highest quintile was associated with higher, but not statistically significant, odds of IHD (OR: 2.04 Asian Indian, and OR: 4.83 Chinese, OR: 1.37 NHW). When CVD was considered as an outcome, the same elevated but not statistically significant association was observed for each racial/ethnic group (OR: 1.63 Asian Indian, OR: 3.47 Chinese, and OR: 1.28 NHW) (Table 3). Median levels of Lp(a) were higher in Asian Indians with either CVD (64.5 nmoL/L) or IHD (64 nmoL/L) when compared to NHW (30, 32) or Chinese (29, 29) with CVD or IHD, respectively.

Sensitivity analyses were performed by including the subset of patients who were treated with niacin. Of those who had Lp(a) measured, 14.6 % (423) received a prescription for niacin. A niacin prescription was more common among men compared to women (18.7% versus 8.3%, *P* < .0001) and more common in patients with abnormal Lp(a) (>75 nmol/L) compared to normal Lp(a) (11.9 versus 6.0%, *P* < .0001). When niacin use was included in logistic regression models modeling CVD outcomes adjusting for the same aforementioned covariates, there was a positive, but still not statistically significant, association between niacin use and CVD outcomes. There was a similar positive, not statistically significant, association between niacin use and IHD outcomes.

## 6. Discussion

In this multiethnic study, our major finding was that Lp(a) levels differed between NHW, Asian Indians, and Chinese. These patterns were similar in men and women; the differences did not quite reach statistical significance in women, but this is likely due to the smaller sample size and larger variability in Lp(a) levels. In addition, we report that Lp(a) levels are associated with a higher prevalence of IHD and CVD in Asian Indians and Chinese although this association, limited by a small number of events, was not statistically significant.

This is not the only study to show that Lp(a) levels differ significantly between these three racial/ethnic groups, with investigators in Singapore also finding higher Lp(a) levels in Asian Indian than Chinese newborns [16] and the SHARE study in Canada finding higher Lp(a) levels in Asian Indians than Chinese adults [15]. However, the SHARE study also found significantly higher Lp(a) levels in Asian Indians than NHW, while a previous study from Michigan found that Asian Indian women exhibited significantly higher levels of Lp(a) than NHW women [24]. While our study corroborates the significant difference in Lp(a) levels between Asian Indians and Chinese cohorts, the discrepancy between our results and previous results regarding Lp(a) levels in NHW and Asian Indians has two possible explanations. First, the discrepancy could be due to a lower sample size of Asian Indians in our analysis compared with previous studies. However, our study included more Asian Indian women than the Michigan study and a similar number of Asian Indians compared to SHARE, making this hypothesis unlikely. Alternatively, the assay used in our investigation, which does not depend on Lp(a) isoform, could have reduced bias in the determination of Lp(a) levels compared to previous assays, which can overestimate Lp(a) levels in the presence of certain Lp(a) isoforms. The assay used in this study may lead to less variation in Lp(a) levels, thus attenuating differences found previously. 

Others have noted an association between high levels of Lp(a) and risk of IHD, particularly investigations that used Lp(a) assays that are independent of isoform size and that measured Lp(a) shortly after venipuncture. In these studies, the risk for IHD due to elevated Lp(a) levels has ranged from 1.4 to 1.6 in predominately NHW cohorts at the 80th percentile of Lp(a) [5, 6, 12]. Gambhir et al., using a case-control design, found that Asian Indians with known coronary artery disease (CAD) exhibited higher median Lp(a) levels than controls [25]. In addition, both cases and controls with a family history of CAD had higher Lp(a) levels than those without a family history of CAD. One weakness of this study was that the Lp(a) assay used was not independent of apolipoprotein (a) size.

We similarly observed an increased risk of IHD for those with higher Lp(a) levels (inclusion in the top quintile) in our cohort of Asian Indians, Chinese, and NHW, but the association was not statistically significant. The lack of statistical significance may be due to the low occurrence of IHD (265 cases) in our cohort; results may differ in larger cohorts or those with a higher prevalence of IHD and CVD. High Lp(a) levels may account for a significant portion of the attributable risk for CVD in Asian Indians given the particularly high levels of Lp(a) in Asian Indians with CVD that we found, as compared to NHW and Chinese. If these results are corroborated in larger studies, Asian Indians with high Lp(a) levels may represent a group with particularly high risk for CVD and should receive aggressive risk factor reduction.

Niacin was frequently prescribed in this cohort of patients with Lp(a) measured, and niacin prescription was correlated with abnormal Lp(a) values. Niacin is the only agent that has been shown to reduce Lp(a) levels. However, this treatment was unequally applied by gender in our cohort, with a higher proportion of men receiving niacin. Such disparities need to be addressed if large-scale clinical trials find a reduction in CVD in patients with elevated Lp(a) level treated with niacin.

Strengths of our study include the assay used, which is independent of Lp(a) isoform, determination of Lp(a) levels without long storage periods requiring freezing and thawing, and a large representation of Asian Indians and Chinese race/ethnicities.

Limitations include the cross-sectional analysis of existing clinical data, which limits conclusions regarding measures of association. There may have been selection bias such that patients who were perceived to be at higher risk for CVD events (i.e., Asian Indians) were sent for Lp(a) testing. There may also have been reverse causation, where those with CVD were more likely to have Lp(a) measured, although this would not be expected to produce higher Lp(a) levels in those with CVD compared to those without who were also measured. A major limitation was the relatively low number of cardiovascular events, especially in the Asian Indian group (which was significantly younger), limiting the power of the study.

## 7. Conclusion

In conclusion, this cross-sectional analysis suggests that Lp(a) levels differ significantly among Asian Indian, Chinese, and Non-Hispanic White subgroups. The association between Lp(a) and cardiovascular outcomes may differ by race/ethnicity as well, although we did not have a sufficient number of CVD events to confirm this. Further studies should strive to disaggregate racial/ethnic groups and stratify by gender when examining Lp(a) among Asians. If replicated, our study suggests that determination of Lp(a) levels may be particularly important in Asian Indians for the prevention of cardiovascular disease.

## Figures and Tables

**Table 1:** Patient characteristics by race/ethnicity and gender.

	Male			Female		
	NHW	Asian	Chinese	NHW	Asian	Chinese
		Indian			Indian	
N	1,126	213	93	896	82	58
Age (years)^M,F^	54.1(13.2)	43.1 (8.6)^‡^	51.5 (12.7)	56.7 (12.4)	44.2 (8.7)^‡^	55 (13.2)
BMI (kg/m^2^)^M^	27.0 (3.8)	25.9 (3.7)	25.4 (3.5)	25.9 (4.7)	26.1 (4.7)	23.3 (3.0)
Lipids (mg/dL)						
Total cholesterol^F^	200 (40)	199 (35)	199 (41)	213 (43)	187 (31)^‡^	218 (40)
LDL cholesterol	123 (35)	123 (30)	120 (37)	124 (38)	110 (27)	129 (36)
HDL cholesterol^MF^	52 (13)	45 (10)^‡^	51 (12)	66 (18)	53 (13)	65 (17)
Triglycerides^M^	126 (76)	156 (82)	158 (113)	114 (70)	117 (55)	120 (67)
Prevalence (%, events)						
Diabetes	5.6% (63)	9.4% (20)	6.5% (6)	7.2% (64)	19.5% (16)	10.3% (6)
Cardiovascular disease^M^	19.4% (218)	6.1% (13)	17.2% (16)	14.2% (127)	3.7% (3)	6.9% (4)
Ischemic heart disease	14.5% (163)	5.2% (11)	15.1% (14)	8.0% (71)	2.4% (2)	6.9% (4)
Stroke	6.2% (70)	0.9% (2)	4.3% (4)	5.2% (46)	0.0% (0)	0.0% (0)
Peripheral vascular disease	3.6% (40)	0.0% (0)	2.2% (2)	4.8% (43)	1.2% (1)	1.7% (1)

All continuous variables listed as mean (SD).

NHW: Non-Hispanic White.

^M,F^Kruskal-Wallis or Chi-square test *P* < .0001 for men or women, respectively.

^‡^Brown-Mood *P* < .0001 for significant difference in median between this group and each of the other two racial groups. Both pairs of comparisons are significant.

**Table 2:** Lp(a) summary results by sex and race/ethnicity.

	Male			Female		
(N)	NHW (1126)	Asian	Chinese (93)	NHW (896)	Asian	Chinese (58)
		Indian (213)			Indian (82)	
Lp(a) (nmol/L)						
Median^M^	27	35	19	34	41	27
Percent in highest quintile	18%	22%	10%	24%	26%	14%
Percent with Lp(a) > 75	22%	26%	11%	28%	28%	19%

NHW: Non-Hispanic White.

The highest quintile of Lp(a) was defined for Lp(a) ≥ 89 nmoL/L.

^M,F^Kruskal-Wallis or Chi-square test *P* < .0001 for men or women, respectively.

No group was significantly different than the other two by pair-wise Brown-Mood tests of the median at *P* < .0001.

**Table 3:** Multivariate adjusted odds ratio for association between Lp(a) (top quintile versus bottom 4 quintiles) and CVD outcomes.

	NHW	Asian	Chinese
		Indian	
Outcome (OR, 95% CI)*			
CVD	1.28 [0.80, 2.05]	1.63 [0.30, 9.00]	3.47 [0.24, 49.23]
IHD	1.37 [0.79, 2.37]	2.04 [0.31, 13.35]	4.83 [0.31, 75.30]

*Adjusted for age, gender, BMI, LDL-C, and HDL-C.
